# Research on Alzheimer's syndromes is critical to improve diagnosis, patient management and non-pharmacological treatments, but is under-pursued

**DOI:** 10.3389/fnagi.2022.1039899

**Published:** 2022-10-17

**Authors:** Carlo Abbate

**Affiliations:** IRCCS Fondazione Don Carlo Gnocchi, ONLUS, Istituto Palazzolo, Milan, Italy

**Keywords:** logopenic, posterior cortical atrophy, frontal-executive phenotype, signs and symptoms, biomarkers

## Introduction

Following the call to arms toward a biological definition of Alzheimer's disease (AD) (Jack et al., 2018), research in the field seems to be dominated by biomarker research (Blennow and Zetterberg, 2018; Zetterberg and Bendlin, 2021; Teunissen et al., 2022). This letter because, beyond the undoubted value of the study of biomarkers, I fear that this call to arms may further orphan an already poorly followed line of research by researchers. This is the study of AD syndromes or clinical phenotypes, which are the complex patterns of signs and symptoms which include cognitive, behavioral, neuropsychiatric, affective, emotional, and motor features exhibited by patients. Therefore, in an effort to counter the current supremacy of pathology and biomarkers in AD research, I felt the need to summarize and emphasize the potential benefits and positive consequences, especially for patients and their families, of AD syndromes research.

## The research on Alzheimer's syndromes is the Cinderella of the field

There is no doubt that research on Alzheimer's syndromes is the Cinderella of the field. Suffice it to say that the first reports of amyloid plaques, the main biological entity of AD, by Alois Alzheimer and Oskar Fischer date back to 1907 (Alzheimer, 1907; Fischer, 1907). Some AD syndromes, however, were first described many decades later and then accepted into current nosography only recently. For example, the term posterior cortical atrophy (PCA) was coined in 1988 (Benson et al., 1988), and the first diagnostic criteria for PCA syndrome were proposed by single-center research groups only in 2002 and 2004 (Mendez et al., 2002; Tang-Wai et al., 2004). The term logopenic was already used in 1990 (Weintraub et al., 1990), but logopenic syndrome was more clearly recognized in 2003 and 2004 (Kertesz et al., 2003; Gorno-Tempini et al., 2004). Finally, fronto-executive syndrome (Ossenkoppele et al., 2015) was first suggested in 1996 (Binetti et al., 1996) and recognized more clearly in 1999 (Johnson et al., 1999). Moreover, it is surprising to consider that we still do not know exactly how many and what are all the clinical pictures that AD can manifest (phenotypic heterogeneity), despite AD being the most common and widespread dementia in the world. Indeed, there are many reports in the AD literature of phenotypes that are quite different and distinct from the currently accepted clinical syndromes (e.g., focal temporal variant, non-fluent aphasia, semantic dementia syndrome, confabulating phenotype, and so on) (Lam et al., 2013; Abbate et al., 2016; Kawakatsu et al., 2021). In addition, my colleagues and I have studied one of these possible unrecognized phenotypes, right-AD (RAD) (Abbate et al., 2019a,b, 2021a), and found that the lack of agreement on the syndrome's occurrence seems to stem more from a lack of research and heterogeneity among studies than from negative findings (Abbate et al., 2019b). Finally, I make a brief test here that will further demonstrate the lack of interest in the study of AD syndromes. All readers reading this are surely familiar with amyloid plaques and the fact that Alois Alzheimer described them in the brain of a patient in 1907 (Alzheimer, 1907). Many, but not all, probably know that amyloid plaques were best described the same year as Alois Alzheimer in 12 patients by Oskar Fischer (Fischer, 1907; Goedert, 2009). However, only a few probably noted that the clinical diagnosis of those 12 patients was presbyophrenia. Finally, I am sure that only a few know that presbyophrenia was an amnesic syndrome quite different from the classic AD amnesic syndrome and similar to Korsakoff's syndrome (Berrios, 1985, 1986; Zervas et al., 1993).

On the other hand, I am not surprised to recognize that the study of AD syndromes has been a Cinderella in the field. In fact, accurately delineating a dementia syndrome requires multidimensional, multispecialty, and sometimes longitudinal assessments that require lengthy collaboration from patients and family members. In addition, research on dementia syndromes needs close collaboration with clinicians, or is carried out by dementia specialists who are both clinicians and researchers. However, there are very few dementia specialists (Hlavka et al., 2019). Moreover, the current mantra of time constraints and affordability of the health care system in many countries penalizes the lengthy and multidimensional visits that are essential to delineate a complex dementia syndrome. A further reason probably stems from the failure of the classical clinic-pathological method in degenerative dementias. In fact, the same dementia syndrome may originate from different diseases (clinical anatomical convergence). For example, posterior cortical atrophy syndrome can be indifferently associated with AD, corticobasal degeneration, Lewy bodies disease, as well as a non-degenerative disease (Creutzfeldt-Jakob disease) (Crutch et al., 2017). Thus, it is sometimes difficult to predict dementia disease from the observed syndrome and some skepticism may emerge about the need for lengthy and accurate syndrome assessments. Finally, delineation of dementia syndromes is ultimately based on simple observation and measurement of patients' behaviors. This method inevitably sounds time-consuming, qualitative, inaccurate and outdated compared to the speed, diagnostic power, accuracy and technological appeal of the advanced instrumental examinations currently available in the dementia diagnostic workup. Moreover, some of the current instrumental examinations can directly demonstrate the presence of dementia disease in vivo, thus supporting an etiological diagnosis rather than a simple syndrome diagnosis.

## Discussion: The reasons why the study of Alzheimer's syndromes remains valuable and unique

The study of AD syndromes remains crucial and irreplaceable for the following numerous reasons (Table 1). First, because of the phenotypic heterogeneity and scattered deposition of amyloid-β in the brain in AD, preclinical detection of the disease does not allow prediction of which clinical features an individual patient will manifest. Second, it is the first appearance of clinical features that establishes the onset of the disease, from many perspectives, clinical, psychological, administrative, legal, and social. Third, the study of a dementia syndrome could prove to be a powerful diagnostic tool. In fact, to detect a dementia syndrome, experienced clinicians have used pattern recognition that allows them to readily recognize complex patterns of behavioral and cognitive characteristics from the detection of a few signs and symptoms (Abbate et al., 2020, 2021b; Johnson et al., 2021), even without or long before the full pattern has manifested. Fourth, it is the anatomy that determines the dementia syndrome (Weintraub and Mesulam, 2009), so an accurate definition of the syndrome could suggest which regions are affected by brain degeneration. This fact could not only help the diagnosis but also guide subsequent brain imaging toward a more targeted examination. Fifth, it is the syndrome that determines the functional impact, as shown by some studies that report worse functional consequences based on the specific AD syndrome (e.g., PCA vs. classic amnestic AD, Shakespeare et al., 2015), or the subtype of MCI (Jekel et al., 2015). Sixth, it is the dementia syndrome that informs how we can interact with the patient, what adaptations need to be made in the patient's home, what cognitive domains need to be stimulated and rehabilitated, and what aids selected. Seventh, the assessment of the course of dementia and the clinical effectiveness of any treatment is based on the repeated evaluation of some clinical features of the syndrome, aimed at detecting some changes over time. Eighth, the careful study of dementia syndrome has made it possible to discover the phenotypic variants of AD, with obvious clinical advantages for diagnosis, management and treatment. At the same time, it helped establish the concept of clinical anatomical convergence in dementia (Seeley, 2017). In turn, the comparison of similar dementia syndromes due to two different diseases on the basis of clinical anatomical convergence has made it possible to identify some subtle clinical differences that could become useful clinical markers of pathology. For example, corticobasal syndrome due to AD was found to be associated with a younger age of onset, the presence of myoclonus, the absence of tremors, more severe visuospatial deficits, the absence of orofacial apraxia, and the presence of dysgraphia than corticobasal syndrome due to corticobasal degeneration (Hu et al., 2009; Burrell et al., 2013). In addition, the differences in syndromes between patients with the same damaged brain regions or the same pathology burden helped to establish the important concept of reserve (Stern, 2009). Finally, I believe that the study of dementia syndromes has promoted and supported the study of neural networks in dementia, likely contributing to the establishment of network theory (Seeley et al., 2009).

**Table 1:** Differential contribution of AD research lines to prevention, diagnosis, management and treatment.

		**Alzheimer's disease and biomarkers^#^**	**Alzheimer's syndromes and clinical assessment^§^**
Prevention	Modifiable risk factors	*	
Diagnosis	Preclinical	*	
	Prodromal	*	*
	Clinical		*
	Anatomical	*	(*)
	Etiological	*	(*)
Management	Psychological impact		*
	Social impact		*
	Functional impact		*
	Administrative issues	(*)	*
	Legal issues	(*)	*
	Course	(*)	*
Treatment	Biological effect (trials)	*	
	Clinical effect (trials)		*
	Cognitive rehabilitation		*
	Aid selection		*
	Home adaptations		*
	Interaction with patients		*

*In parentheses indicate minor roles.

*Without parentheses indicate major roles.

^#^Blennow and Zetterberg (2018); Zetterberg and Bendlin (2021); Teunissen et al. (2022).

^§^Lam et al. (2013); Abbate et al. (2020, 2021b); Johnson et al. (2021); Kawakatsu et al. (2021).

## Conclusion

While the study of biological mechanisms and biomarkers remains fundamental to finding a cure and preclinical diagnosis of AD, the study of AD syndromes is relevant to improving some aspects of diagnosis and unique to improving multiple aspects of non-pharmacological management and treatment. Consequently, this line of research addresses issues directly related to the needs of patients and their families.

## Author contributions

The author confirms being the sole contributor of this work and has approved it for publication.

## Conflict of interest

The author declares that the research was conducted in the absence of any commercial or financial relationships that could be construed as a potential conflict of interest.

## Publisher's note

All claims expressed in this article are solely those of the authors and do not necessarily represent those of their affiliated organizations, or those of the publisher, the editors and the reviewers. Any product that may be evaluated in this article, or claim that may be made by its manufacturer, is not guaranteed or endorsed by the publisher.
